# Five-Year, Disease-Free Survival after Repeat Palliative Multimodality Therapy in a Patient with Recurrent Metastastic Bladder Cancer

**DOI:** 10.1100/tsw.2007.283

**Published:** 2007-10-22

**Authors:** Sebastian Fetscher, Jan Schmielau, Wolfgang Schulze-Seemann

**Affiliations:** ^1^ Division of Hematology and Oncology, Department of Internal Medicine, Sana Hospital Lübeck, Kronsforder Allee 72-74, D-23560 Lübeck, Germany; ^2^ Department of Urology Freiburg, University Hospital Medical Centre, D-79106 Freiburg im Breisgau, Germany

**Keywords:** bladder cancer, lymph-node metastasis, palliative surgery, palliative radiotherapy, regionally metastatic bladder cancer

## Abstract

In appropriately selected cases, palliative therapeutic strategies can be adapted to those special features of cancer biographies that indicate an atypical course of disease. Elucidating these features, and adapting multimodal treatment strategies to them, can lead to significantly superior effects when compared to the routine application of conventional treatment algorhythms. A case of regionally metastactic bladder cancer is presented that documents the value of repeat debulking-surgery and repeat radiotherapy leading to unexpected short-term and long-term treatment results.

## INTRODUCTION

Urothelial cancer of the bladder (UCB) is one of the most common and lethal human malignancies affecting more than 250,000 patients annually, about half of which will succumb to the disease. Median survival of untreated patients with locally inoperable or metastatic disease averaged 3–6 months in historical series. While palliative chemotherapy can improve median survival to 8–12 months, long-term survival remains exceptional[45].

## NODE-NEGATIVE BLADDER CANCER

UCB is regarded as curable only when confined to the bladder[24,32,35]. However, even in patients with locally confined, node-negative bladder cancer treated with radical cystectomy, cure rates vary considerably depending on tumor stage, size, histological classification, and other molecular risk factors[7,24,32,35].

## BLADDER CANCER WITH PELVIC LYMPH NODE INVOLVEMENT

Patients with node-positive UCB have a worse outcome than those with node-negative disease. Whether these patients should be treated with radical cystectomy and extended bilateral lymphadenectomy or without surgery remains subject to debate; locoregional radiotherapy with chemotherapy has been considered an alternative to surgery in node-positive disease[8,11,12,13,14,15,21,29,36]. Regardless of treatment chosen, however, long-term cure is attained only by a small minority of node-positive patients[7,8]. For example, median survival for patients with more or less than five positive nodes after complete tumor resection was 15 and 27 months, respectively, in a Swiss study[7,21].

The following factors have been shown to influence disease-free and overall survival in patients with node-positive UCB: T- and N-stage, density (defined as ratio of positive nodes to total number of nodes examined) and extent of nodal disease (defined by cut-offs at <5, 6–7, and >8 affected nodes), extracapsular carcinomatous extension, and organ-confined or nonorgan-confined primary tumors[7,12,14,21,35,36,42].

The number of lymph nodes resected in UCB reflects the radicality of the surgical approach, physiological variation in nodal anatomy, and differences in the diligence of pathology examination. As in other solid tumors, such as breast or colon cancer, the number of resected nodes has been shown to have an impact on survival even in node-negative tumors[14,36]. In node-positive disease, however, a statistically significant relationship between number of lymph nodes examined and survival could not be confirmed by most investigators[12,14,36,37].

The recommended number of pelvic lymph nodes to be resected with radical cystectomy and extended bilateral lymphadenectomy is said to be between 20 and 25[7]. For patients with five or fewer positive nodes, a survival benefit was demonstrated when a minimum of 16 nodes was resected in total[14]. Reduction in the number of pelvic nodes resected at the time of primary operation in locally advanced urothelial cancer of the bladder (UTCA) resulted in inferior staging and inferior relapse-free survival in a recent study[4a]. These observations indicate that lymphadenectomy in UCB is not merely a staging procedure, but may have a therapeutic and prognostic role in selected populations[5a,7,12a].

## BLADDER CANCER WITH EXTRAPELVIC, INTRA-ABDOMINAL NODAL INVOLVEMENT

Radical cystectomy with bilateral pelvic lymphadenectomy can have a curative role in selected patients with pelvic lymph node disease; in particular, postchemotherapy surgery has been shown to result in long-term, disease-free survival in some patients when adequate measures to select surgical candidates after response to palliative chemotherapy were taken[5a,12a].

The question, whether patients with extrapelvic nodal disease – in the absence of visceral or other metastases – may benefit from surgical and other treatment modalities has not been addressed. Median survival of patients with untreated extrapelvic nodal disease has been reported at 6 months[45]. With the use of palliative chemotherapy or radiotherapy, median survivals of up to 12 months have been described[45].

Chemotherapy with methotrexate, vinblastine, adriamycin and cis-platinum (MVAC) or gemcitabine plus cis-platinum (GC) in patients with locally advanced and metastastic UCB resulted in a 5-year overall survival of 6.8 and 20.9% for patients without or with visceral metastasis, respectively[48]. However, a third of the patients in this study did not have metastatic disease (defined as M1 by Union International Contre Cancer (UICC) criteria), so that the survival of patients with M1-stage by virtue of extraregional lymph node metastasis probably is worse than these figures.

## BLADDER CANCER METASTATIC TO EXTRANODAL SITES

Overall median survival for patients treated with GC is 12–14 months; estimated 4-year survivals range from 5–15%. Results for patients treated with MVAC are comparable. Karnofsky performance score (KPS), presence or absence of visceral metastases, elevated serum alkaline phosphatase, as well as the number of disease sites (>3 vs. ≤3) have been described as prognostic variables[13,14,15].

A Swiss group recently reported a cumulative 17% 5-year survival rate from MVAC studies. Pretreatment prognostic factors significantly affect survival. Patients with a poor KPS and evidence of visceral disease did not experience long-term survival, in contrast to a 33% likelihood of 5-year survival in patients with none of these features[7,21].

## CASE REPORT

In April 1998, a 61-year-old patient experienced relapsing urinary tract infections. Infections were treated. No structural abnormalities of the urinary tract were identified, however, atypical cells were observed in urine cytology. Follow-up cytologies from 1998 to 2001 displayed an increasing extent of atypical and dysplastic cells. In July 2001, cystoscopy revealed a small superficial bladder malignancy. Repeat cytoscopies led to the discovery of relapse lesions, leading to repeat transurethral resections between July and November 2001. Tumor stage progressed from pTis, pTa, pT1, to pT2, while grade progressed from GIIb to GIII during this time.

In December 2001, repeat cystoscopy documented muscle-invasive carcinoma. While abdominal computed tomography (CT) did not reveal extravesical disease, magnetic resonance investigation (MRI) of the pelvis revealed left iliac, right inguinal, and retroperitoneal lymph node enlargement. After discussion of the therapeutic options with the patient, the decision to perform a radical cystoprostatovesiculectomy with bladder reconstruction using an ileum conduit was made. Intraoperatively, lymph node metastases as demonstrated by MRI were detected and resected by radical bilateral pelvic lymphadenectomy; included were the following nodal compartments: iliaca interna, iliaca externa, obturatoria; limit of initial nodal resection was the aortic bifurcation. However, when surgical inspection documented extension of lymph node spread to the left para-aortic region not visualized by MRI, lymphadenectomy was extended to this area; resection was limited to 1 in. above the inferior mesenteric artery. Postoperative tumor stage was pT3b, pN2 (07/14), pM1 (extraregional lymph nodes; 5/8 nodes positive), G3, R0.

In January 2002, adjuvant chemotherapy with cis-platinum and gemcitabine was given. However, due to severe side effects, treatment was terminated after a single infusion of these agents. Therapy with cis-platinum resulted in extensive intraperitoneal fluid accumulation, presumably by leakage through the neobladder. The resulting aseptic peritonitis with subileus was treated with intensive medical management. After recuperation from this WHO IV complication, gemcitabine was given, however, after the infusion WHO II, capillary leak syndrome ensued[4]. Lastly, third-line adjunct chemotherapy using a combination of carboplatinum and vinblastine resulted in the rapid development of WHO II peripheral neuropathy. Based on these negative experiences, no further chemotherapy was given to the patient since.

In July 2003, CT showed enlarged lymph nodes in the para-aortic space. At the time of this, and of each subsequent relapse until 2007, the patient was asymptomatic. Salvage lymphadenectomy demonstrated metastatic involvement in 12 of 21 nodes. In view of negative prior experience with chemotherapy, external beam radiation therapy (37.8 Gray) was given as adjunct to surgery.

In March 2004, CT and positron emission tomography (PET) revealed left para-aortic and left cervical node enlargement. Needle biopsy of the cervical node demonstrated cells compatible with urothelial carcinoma. With metastatic disease above the diaphragm, radiation therapy was chosen as palliative. Treatment was performed in Brussels using the NOVALIS technique, which applies stereotactic planning and application of external beam-focused radiation analogous to intracranial stereotactic approaches. 30 Gray were applied to left cervical and para-aortic region.

In May 2005, CT and PET results again indicated para-aortic disease. A second salvage lymphadenctomy demonstrated 6 of 14 nodes to be affected; for the first time, the procedure was classified as R1 resection in two areas. Surgical exposure included the entire length on the aorta below the diaphragm, including the pararenal space. Extensive carcinomatous plaques tightly adherent to the aortic wall were resected; this manifestation had not been detected by CT or PET. Again, adjunct radiotherapy (39.6 Gray) was performed using the above-mentioned NOVALIS technique.

As of March 2007, 5 years and 3 months after the diagnosis of a pT3b, pN2, pM1, G3 UCB, signs or symptoms of persistent or recurrent disease were absent. The patient works full time as an academic teacher and president of an international postgraduate institution. He has missed an average of 2 weeks of work per year since 2001. His quality of life has been affected only with regards to altered urogential function. No other significant therapy-related side effects have occurred. In particular, despite the cumulative resection of 57 retroperitoneal lymph nodes during three lymphadenectomies, secondary lymphedema of the lower extremities has not been observed.

Since the patient's treatment history began at the age of 64 and included four transurethral resections, three extensive abdominal operations, two chemotherapies, and three full-dose courses of radiation therapy, the absence of debilitating treatment sequelae may be as remarkable in this case as is the disease-free state as of March 2007 by clincial, CT, and PET criteria 5 years after the initial diagnosis of UCB.

## Addendum (October 2007)

In April 2007, the patient developed symptomatic, right-sided ureteral obstruction necessitating implantation of a double-J cathether. After CT documentation of ureteral tumor invasion, salvage lymphadenctomy #3 was performed demonstrating metastatic disease in 7 of 14 resected lymph nodes and in retroperitoneal soft tissue; for the first time, the procedure had to be classified as R2 resection. Ureteral obstruction was relieved, the stent could be removed, no symptoms remained.

Based on these intraoperative findings, a gene-expression analysis of the tumor tissue (Onctest Company, Freiburg, Germany; chief investigator, H.H. Fiebig) was performed and resulted in the recommendation to apply systemic therapy with bevacizumab and docetaxel. After 4 cycles of this therapy, a partial remission of residual intra-abdominal disease was observed. Chemotherapy and antiangiogenetic therapy are planned to continue for another 2 months. Afterwards, maintenance treatment with bevacizumab is envisioned.

## DISCUSSION

The case history reported here contains a number of unusual features that deserve discussion. To begin with, the sequence of treatments chosen for this patient reflect an unusual constellation of elements.

- An unusual pattern of regionally metastastic and relapsing UCB, restricted to nodal disease during a period of more than 5 years
- Relapsing retroperitoneal nodal metastases unusually amenable repeated surgical resection
- Residual disease after each operation apparently sensitive to adjunct radiation therapy
- A patient with an unusually high tolerance for repeated surgical and radiation therapies
- A patient with a particular intolerance to chemotherapy, preventing the application of adequate chemotherapy or chemoradiotherapy prior to or after the surgical procedures
- A urological surgeon able to perform four sequential abdominal lymphadenectomies with the initial cystoprostatovesiculectomy, as well as 19, 41, and 64 months later without significant sequelae

- A radiotherapy institution performing a palliative primary radiation for first nodal relapse 19 months after initial therapy and another institution using a different technique for palliative radiation of nodal relapses 27 and 41 months after initial diagnosis
- A medical oncologist involved as consultant in each of the above decisions, in particular, in the decision to reoperate nodal relapse.

Given these factors, the clinical observations made in this case cannot be simply translated to the management of other patients with UCB. Nevertheless, the following reflections may deserve attention:

- The case demonstrates that poor-risk patients with regionally metastastic UCB can repeatedly achieve disease control with multimodal therapy despite repeated relapse. This message is all the more important since the patient received pessimistic conventional recommendations all along his patient career. At the initial diagnosis of muscle-invasive UCB, palliative radiochemotherapy was recommended. Since the true extent of nodal metastastic disease was only discovered by primary extensive surgery, this treatment was bound to fail predictably since a part of the lymph nodes involved would have been outside the conventional radiation field.
- The patient's poor tolerance of chemotherapy likely would have made the implementation of an effective radiochemotherapy impossible, at least in the postoperative setting. At each of the relapses, standard recommendation had been palliative chemotherapy using GC or MVAC. Neither repeat surgery, nor repeat radiotherapy was considered a useful option. If these treatment options had been forfeited, as was strongly recommended, disease control as documented here would hardly have been achieved.
- The only alternative for this case suggested by the literature would have been to institute neoadjuvant chemotherapy or chemoradiotherapy in order to improve the results of secondary surgical resection[5a,12a]. The curative potential represented by the favorable results published in the operable, chemosensitive subset of patients with locally advanced or regionally metastastic bladder cancer by Dodd and Herr is, however, by no means universally appreciated by the urological community. The case presented here documents another route to achieve disease control in regionally metastatic bladder cancer: the sequential implementation of lymphadenectomy and postsurgical radiotherapy.

In sum, the case history documented here supports the conclusion that by adapting palliative therapeutic strategies to unique cancer biographies, treatment results can be achieved that may be significantly superior to those attained by executing conventional treatment algorithms. With the ever-increasing pressure on the medical and oncological community to define and adhere to "treatment standards", individual observations such as the one presented here may serve as a reminder that, in properly selected patients, metastastic bladder cancer can be successfully treated with repeat surgery and adjunct radiotherapy.
